# Dehydration of plant cells shoves nuclei rotation allowing for 3D phase-contrast tomography

**DOI:** 10.1038/s41377-021-00626-2

**Published:** 2021-09-15

**Authors:** Zhe Wang, Vittorio Bianco, Daniele Pirone, Pasquale Memmolo, Massimiliano Maria Villone, Pier Luca Maffettone, Pietro Ferraro

**Affiliations:** 1 Dipartimento di Ingegneria Chimica dei Materiali e della Produzione Industriale, Università degli Studi di Napoli “Federico II”, Piazzale Tecchio 80, 80125 Napoli, Italy; 2 NEAPoLIS, Numerical and Experimental Advanced Program on Liquids and Interface Systems, Joint Research Center CNR - Università degli Studi di Napoli “Federico II”, Napoli, Italy; 3 CNR-ISASI, Institute of Applied Sciences and Intelligent Systems “E. Caianiello”, Via Campi Flegrei 34, 80078 Pozzuoli, Napoli, Italy; 4 Dipartimento di Ingegneria Elettrica e delle Tecnologie dell’Informazione, Università degli Studi di Napoli “Federico II”, via Claudio 21, 80125 Napoli, Italy

**Keywords:** Interference microscopy, Imaging and sensing

## Abstract

Single-cell phase-contrast tomography promises to become decisive for studying 3D intracellular structures in biology. It involves probing cells with light at wide angles, which unfortunately requires complex systems. Here we show an intriguing concept based on an inherent natural process for plants biology, i.e., dehydration, allowing us to easily obtain 3D-tomography of onion-epidermal cells’ nuclei. In fact, the loss of water reduces the turgor pressure and we recognize it induces significant rotation of cells’ nuclei. Thanks to the holographic focusing flexibility and an ad-hoc angles’ tracking algorithm, we combine different phase-contrast views of the nuclei to retrieve their 3D refractive index distribution. Nucleolus identification capability and a strategy for measuring morphology, dry mass, biovolume, and refractive index statistics are reported and discussed. This new concept could revolutionize the investigation in plant biology by enabling dynamic 3D quantitative and label-free analysis at sub-nuclear level using a conventional holographic setup.

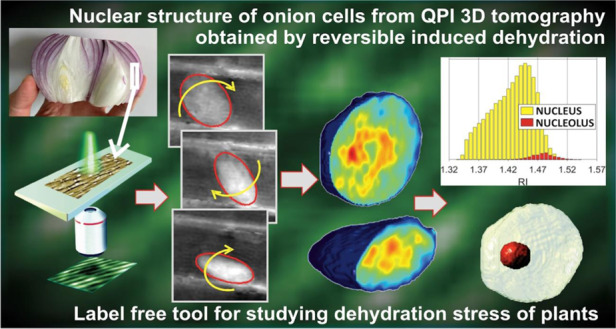

## Introduction

Optical microscopy of transparent biological specimens is mostly governed by two outstanding concepts, namely marker-based and label-free imaging. Both are devoted to induce a contrast mechanism to enable the visualization of all the sample structures that are transparent at the wavelength of the light probe. Marker-based imaging relies on dyes and various contrast agents to excite fluorescence in some sample areas. Specific information can be gained by accurately changing the marker, in order to excite the sole sub-cellular structures of interest. On the other hand, label-free methods are noninvasive and do not require markers, simplifying sample preparation at the cost of specificity, and avoiding any possible toxicity effect of the contrast agent. In particular, quantitative phase imaging (QPI) relies on the phase-contrast between the sample and the surrounding medium to image transparent structures, which in turn is due to refractive index differences^1–3^. Digital holography (DH) in microscope configuration is one of the preferred label-free techniques to image biological samples in the form of confluent cell layers, tissues, and suspended cells in high-throughput microfluidic streams. DH gives access to the sample complex amplitude, from which the phase-contrast map can be measured^4–8^. The digital hologram of the sample can be captured out-of-focus, and then digitally refocused in post-processing by numerically solving the diffraction integral that simulates the process of wavefront propagation. DH is well assessed and has been deeply exploited in all fields of biology, diagnostics and medicine, microfluidics and lab-on-a-chip imaging, 3D tracking, cell mechanics, point-of-care testing, environmental monitoring^4–14^. In DH, the measured quantitative information is the optical thickness contrast, which is the integral product between the sample refractive index contrast and its physical thickness along the optical axis. In order to decouple this information, multiple channels have to be exploited, e.g., multiple wavelengths or illumination angles^15^. In particular, phase-contrast tomography (PCT) probes the sample from different directions to achieve resolving capability along the optical axis and to measure the sample refractive index 3D distribution^16–22^. Multi-angle illumination can be realized by moving the light source with respect to the object^16,17^. Recently, in-flow cytotomography has been demonstrated by inducing controlled sample rotations in microfluidic streams, which is advantageous to maximize the throughput^22^. The latest advances in PCT based on QPI, and the recent harmonic optical tomography (HOT) approach have extended the variety of specimens that can be analyzed, including nonlinear and inhomogeneous objects as well as strongly scattering specimens, paving the way to the tomographic analysis of model organisms based on QPI^23–26^.

**Fig. 1:**
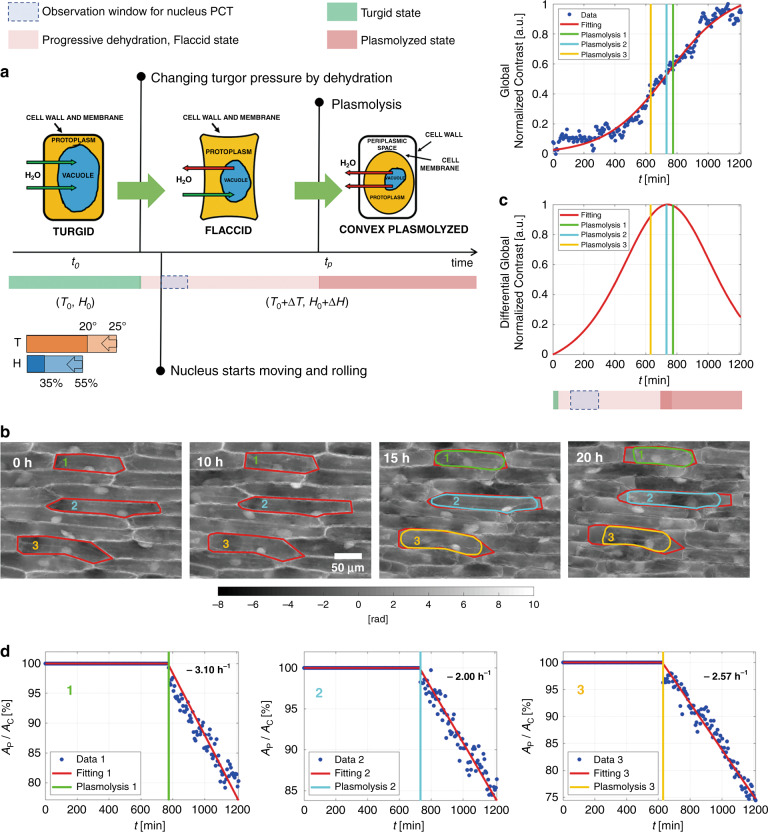
(Supplementary Movie 1) Exploiting induced dehydration in onion epidermal cells. **a** Sketch of the induced change in a cell turgor pressure due to the cell aqueous content. Cell dehydration can be induced acting on the environmental temperature, *T*, and Humidity, *H*. The released nucleus is free to move and rotate. Dehydration also allows imaging the cell with enhanced phase contrast, thus boosting the optical system sensitivity. Within the observation window, nucleus rotation is exploited for PCT. **b** Four QPMs at different times with the cell walls highlighted in red and the corresponding cell membranes after the start of plasmolysis shown in green, cyan, and yellow. **c** Top: global image contrast normalized between 0 and 1, with the logistic fitting overlapped in red. Bottom: differential global normalized contrast, i.e., the time derivative of the logistic function, whose peak marks the breaking plasmolysis event. The vertical lines correspond to the plasmolysis times, i.e., *t*_*P1*_ = 12.9 h for the first cell, *t*_*P2*_ = 12.2 h for the second cell, and *t*_*P3*_ = 10.5 h for the third cell. **d** For each cell, the temporal trend of the percentage ratio between the protoplast area, *A*_*P*_, and the cell area, *A*_*C*_, with a linear fitting overlapped in red. Values at the top right corner are the angular coefficients of the linear fitting after the plasmolysis event

Several works have been proposed over the years to enhance the performance of DH imaging, e.g., aimed at increasing spatial and temporal resolution, field of view (FoV), depth of focus, and signal-to-noise ratio^10,27–29^. However, due to the limited sensitivity of any optical system and the intrinsic sensor quantization, our capability to observe tiny inner structures of the sample depends on the refractive index contrast itself. In the case of thin tissue slices, confluent cell layers, or cells spread onto LoC substrates, the refractive index plays a major role in determining the observed phase-contrast. In the absence of an adequate refractive index difference between the structure of interest and the surrounding medium, even high spatial resolution is not sufficient to guarantee neither a correct quantitative phase-contrast mapping nor tomographic imaging. In order to improve the phase contrast, the use of dyes or the intracellular injection of glycerol, genetically encoded agents, or other strategies for DH microscopy have been proposed^30–32^. Here we deliberately induced dehydration in epidermal onion cells and exploit it to achieve a more convenient imaging condition in 3D. In fact, we show that dehydration provokes the progressive loss of intracellular water content inducing rotation of the cell nucleus over a wide range of angles thus permitting us the accomplishment of 3D imaging by PCT without any mechanical or electro-optical laser beam scanning device. We studied the dehydration process through DH time-lapse experiments and we determined an optimal time window to observe the sample before plasmolysis starts. In other words, the process we have induced is reversible within the selected time-window and thus can be used to improve 3D imaging by PCT in a nondestructive manner. We demonstrate in the following that the concept of controlled dehydration allows us to access the nucleolus of onion epidermal plant cells and to measure its 3D properties in simple mode. Plant cells contain the vacuole, i.e., a roundish tank surrounded by the tonoplast membrane. In a mature plant cell, the vacuole occupies between 80 and 90% of the internal cell volume and is responsible for turgor pressure. Turgor pressure gives solidity to the cell and is generally determined by the water content of the vacuole (see Supplementary Information). By controlling the environmental temperature and humidity, we alter the cell turgor through the variation of its aqueous content, as sketched in Fig. 1a. Moreover, we make use of 3D finite-element direct numerical simulation (DNS) to furnish a simplified description of the fluid dynamics inside onion epidermal cells during dehydration. Results show that, upon the dehydration process starts, curved streamlines develop in the liquid inside the cell. This explains at glance why the nucleus begins to rotate, as clearly observed in our experimental tests. At the same time, dehydration also leads to a rearrangement of the cytoskeletal structures and to a relaxation of the mechanical constraints that keep the nucleus fixed in its initial position^33–36^, thus promoting its rotation. Here we demonstrate that, when specific conditions are met, it is possible to exploit this induced rotation to accomplish PCT of the nuclei. It is important to underline that the rotations experienced by the nuclei occur spontaneously, they are unknowns of the problem we tackle, and the corresponding angles are difficult to be measured. Nonetheless, thanks to the unique 3D imaging features of a digital holographic microscope, we show that it is possible to retrieve accurate 3D positions and angles of the nuclei during their rotation^37,38^. For this purpose, we develop an ad hoc angle tracking algorithm to estimate the rotation angle, which is unknown but essential for any tomographic reconstruction. Finally, from the 3D tomograms, we identify the nucleolus and, for the first time to the best of our knowledge, we measure its refractive index, dry mass, 3D morphological features, and bio-volume with a label-free and non-invasive method. More conventional systems for operating PCT of cells by angular mechanical scanning exist and technology is mature enough to provide them in the form of commercial microscopes^16–19^. In principle, these could be used to infer information on the nucleus as well. The main advantage of the proposed method over any system that operates an angular scanning of the light probe with respect to the sample is the possibility to access a much wider range of angles, since the full rotation of the nuclei can be exploited. This principle follows the same idea of tomographic flow cytometry, where the cell rotation is exploited while keeping the system fixed^22^. Another advantage is the higher simplicity of the imaging system that does not rely on rapidly moving/rotating parts. Instead, once angle tracking is performed, any conventional holographic interferometer can serve as a tomographic phase-contrast microscope for this specific application. This is obtained at the cost of a longer acquisition time, which is a drawback of this method over conventional tomographic microscopes (it will be shown in the results section that PCT is obtainable in less than four hours in our experiments). Plant cells’ taxonomy, physiology, reproduction, and evolution are topics deeply studied in all fields of botany and agriculture, ecology and animal biology, pharmacology and medicine, food production, and smart farming. The actin cytoskeleton of plant cells is known to play a key role in the morphogenesis and function of highly specialized cell types, and to drive cytoplasmic streaming^39,40^ and mitochondrial movement^41^. Thus, it is of interest to dynamically track 3D intracellular movements and cell trafficking processes with the transport of biological material. In particular, imaging onion cells is convenient since the onion epidermis shows large transparent cells in a monolayer and exhibits a clearly identifiable mitotic process^42,43^. On the other hand, because of their high number of rRNA genes and the large size of the dense fibrillar component mass inside the nucleus, onion cells are the most appropriate samples for studying plant viruses^44^ and immunolocalization^45^. It is worth remarking that this simple strategy we propose is fully reversible as we are able to observe wide-angle rotation before the plasmolysis event. Thereby, the dehydration process can be stopped before the cells experience irreversible damages. This means the sample could be brought back to its normal healthy state by reversing the dehydration process after the tomographic shooting.

## Results

### Phase-contrast optimization by induced dehydration

We performed a long-time lapse of an onion epidermal cells’ layer by a holographic microscope in an off-axis configuration. We recorded 203 holograms, one every 6 min, using the experimental DH setup described in the “Materials and methods” section. Thus, we observed the dehydration of the onion cells for about 20 h. For each recorded hologram, we applied conventional DH processing (see “Materials and methods”), and we obtained the corresponding quantitative phase maps (QPMs). In Fig. 1b, from left to right, we show four QPMs taken at times 0, 10, 15, and 20 h, respectively. Moreover, we highlight in red the cell wall of three cells and in green, cyan, and yellow the corresponding protoplast after the plasmolysis has occurred, i.e., at *t*_*P1*_ = 12.9 h for the first cell, *t*_*P2*_ = 12.2 h for the second cell, and *t*_*P3*_ = 10.5 h for the third cell. It is clear that in onion epidermal cells plasmolysis is convex, as reported also in bright field and fluorescence images^46^. During the dehydration, loss of the cell sap and in turn reduction of the turgor pressure occur. We can evaluate the temporal trend of the phenomenon by monitoring the phase-contrast^32^ in the holographic reconstructions. Indeed, the evaporation of the aqueous content makes the image contrast increase. For each QPM, we evaluate the global image contrast as the sum of the modulus of its Laplacian. We report it in the top of Fig. 1c, normalized between 0 and 1. The global normalized gradient is fitted well by a logistic function that is overlapped in red to the measured values. It is reasonable to expect that the plasmolysis takes place when the rate of aqueous loss is maximum. As shown by the vertical lines, the plasmolysis of the three cells occurs in correspondence with the region of maximum slope, i.e., the region in which the growth rate of the image contrast is maximum, as also confirmed by the differential global normalized contrast in the bottom of Fig. 1c. This is reasonable since plasmolysis is a breaking event in the dehydration process that should correspond to the highest increment of the function that describes the loss of turgor pressure. In other words, the logistic function and its time derivative are found here to describe quite well the ongoing dehydration process and how it leads to the plasmolysis. Finally, in Fig. 1d, for each of the three cells, we show the temporal trend of the percentage ratio between the protoplast area, *A*_*P*_, and the cell area, *A*_*C*_. After plasmolysis, these trends are fitted well by a linear function (in red), whose angular coefficient is reported in black for each cell. Supplementary Movie 1 shows the complete time-lapse from which the maps in Fig. 1b are extracted, along with the corresponding analysis shown in Fig. 1c, d.

### Modeling induced dehydration and plasmolysis

**Fig. 2:**
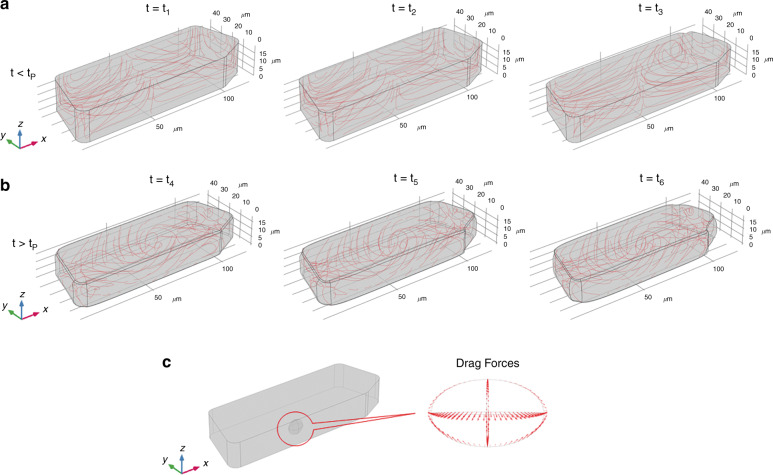
Geometry of the domain considered in finite-element DNS of cell dehydration at increasing values of time. **a** (Supplementary Movie 2) Cell undergoing dehydration, before plasmolysis, and reducing its height along *z*-direction. **b** (Supplementary Movie 3) Cell undergoing dehydration, after plasmolysis, and reducing its lateral sizes along *x*- and *y*-directions. Liquid streamlines are displayed in red in (**a**, **b**). **c** Vector field depicting the drag force acting for *t* *<* *t*_*P*_ on an ellipsoid with dimensions and position comparable to the nucleus of cell 1 in Fig. 1

The cytoskeleton is a network composed of three types of protein fibers, which extend from the nucleus to the cell membrane within the cytoplasm, namely intermediate filaments, microfilaments (or actinic filaments), and microtubules. In particular, the intermediate filaments have a purely structural role because they create a support scaffold inside the cell. Therefore, they avoid the movements of some organelles, including the nucleus. The cytoskeleton has specific biomechanical properties^33^ and it reorganizes itself when the plasmolysis occurs^34–36^. As discussed above, the reduction of cell turgor associated to dehydration makes the intermediate filaments blocking the nucleus relax, thus allowing for its movement. In order to investigate whether fluid dynamic conditions for nucleus rotation might establish during cell dehydration, we performed a 3D finite-element DNS of a simplified system mimicking an onion epidermal cell through the commercial software COMSOL Multiphysics™ V5.5 (details on the mathematical model are given in the Supplementary Information). In Fig. S2, we report the initial geometry of the computational domain, i.e., a prism containing water with a shape similar to cell 1 in Fig. 1. The simulation was divided into two different parts. Supplementary Movie 2 shows the first part in which the simulated cell undergoes dehydration before the plasmolysis event, i.e., *t* *<* *t*_*P*_. In this stage, dehydration was simulated by considering water evaporation through the top surface parallel to the *xy*-plane, whereas the other surfaces of the solid figure were considered to be impermeable. Upon the beginning of dehydration, the cell started deflating, as highlighted in Fig. 2a by the decrease of the cell’s height in the *z*-direction at three increasing values of time before plasmolysis. Supplementary Movie 3 shows the case of a cell that keeps dehydrating after the plasmolysis has started (*t* *>* *t*_*P*_). In this stage, since the cell membrane has detached from the cell wall, as shown in Fig. 1b, the dehydration process was simulated by imposing water evaporation on the cell lateral sides too. Therefore, in Fig. 2b, the lateral sides of the simulated cell are free to move and the cell volume significantly reduces in the reported three successive times after plasmolysis has occurred, following the real trend measured in Fig. 1d. As a consequence of the dehydration, a pattern of curved liquid streamlines (displayed in red in Fig. 2) arises inside the cell and enhances during the analyzed process. This gives a fluid dynamic argument for the observed 3D movements of the cell nucleus during dehydration, also because dehydration induces a rearrangement of the cytoskeleton that weakens the constraints keeping the nucleus fixed. In this regard, it should be remarked that, as the objective of the numerical simulations included in this paper is to verify that fluid dynamic conditions for cell rotation can establish as a consequence of dehydration-due cell deflation, the mechanical model considered for this purpose is very simple and focuses on the liquid contained in the vacuole, thus not including neither the nucleus nor the cytoskeleton. As a further element, we report in Fig. 2c the vector field depicting the drag force acting for *t* *<* *t*_*P*_ on an ellipsoid with dimensions and position comparable to the nucleus of cell 1, showing that such an object would undergo a 3D roto-translation as the experimentally observed ones when affected by the computed fluid dynamic conditions. In conclusion, the nucleus starts to move in 3D and rotate within the cell, thus providing optical access to its 2D projections. This effect can be observed and exploited even before the plasmolysis event, as confirmed by the experimental results in the next sections.

### 3D tracking of the nuclei

**Fig. 3:**
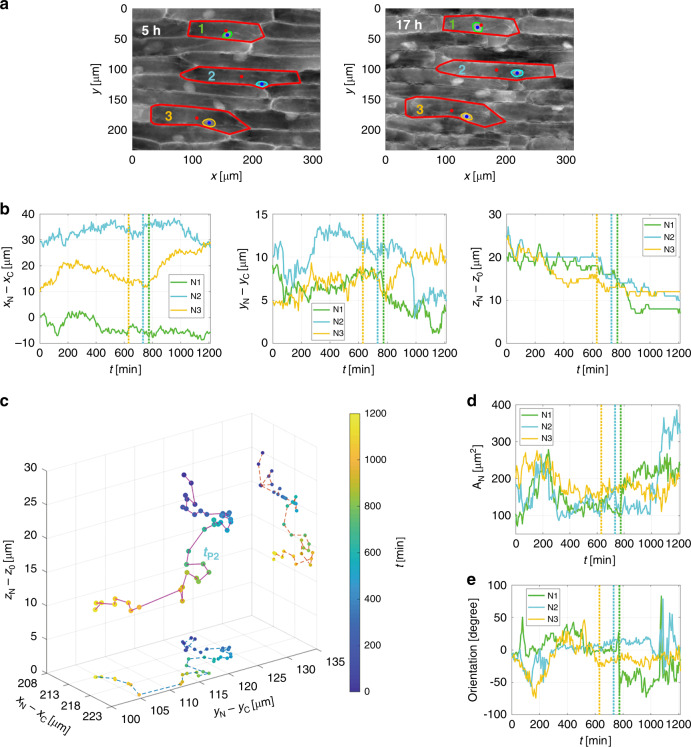
(Supplementary Movie 4) Movements of the nuclei induced by dehydration. **a** Two QPMs at different times with the cell walls highlighted in red and the corresponding nuclear membranes in green, cyan, and yellow. Dots are the centroids of the nuclei (blue) and of their cells (red). **b** Tracking of the three nuclei. From left to right, the first two plots are the nuclear transverse position (*x*_*N*_,*y*_*N*_) with respect to the cell transverse position (*x*_*C*_,*y*_*C*_), while the third plot is the nuclear axial position *z*_*N*_ with respect to the reference axial position *z*_*0*_, which corresponds to the lower surface of the layer of onion cells. **c** 3D tracking of the second nucleus, with the corresponding 2D tracks in the *xy*-and *xz*-planes. As indicated by the colorbar, different dot colors correspond to different times, including the time of plasmolysis *t*_*P2*_ = 12.2 h. For the sake of clarity, we show one position every 30 min, corresponding to a time downsampling of the plots in (**b**). **d** Temporal trend of the area of the three nuclei *A*_*N*_ in the *xy*-plane. **e** Temporal trend of the orientation of the three nuclei with respect to the *x*-axis. In (**b**, **d**, **e**), the vertical lines highlight the plasmolysis of the analyzed cells

In Fig. 3a, we show two typical examples of QPMs observed during cell dehydration, respectively at times 5 and 17 h. We highlight in red the cell walls of three cells and in green, cyan, and yellow the corresponding nuclear membranes. We use the centroid method^37^ to calculate the transverse position (*x*_*N*_,*y*_*N*_) of each nucleus (blue dot) and the transverse position (*x*_*C*_,*y*_*C*_) of the corresponding cell (red dot). For each nucleus, in Fig. 3b we report the transverse positions relative to the centroids of the outer cells. Indeed, as shown in Fig. 3a, cells within the FoV move slightly upwards throughout the recorded sequence. However, the cell wall always retains its initial shape, so the relative transverse positions allow neglecting this effect. Moreover, the holographic technique allows recovering the axial position of a sample by adopting autofocusing criteria, e.g., through the Tamura coefficient optimization^37^. Therefore, in Fig. 3b, we also report the axial position *z*_*N*_ of each nucleus with respect to a reference axial position *z*_*0*_ that corresponds to the lower surface of the layer of the onion cells. In Fig. 3c, we show on the same plot the 3D tracking of the second nucleus, which is a synthesis of the information reported in Fig. 3b. For the sake of clarity, we show one position every 30 min, corresponding to a time downsampling of the plots in Fig. 3b, and we highlight the time the plasmolysis event takes place, *t*_*P2*_ = 12.2 h. Moreover, we report the corresponding 2D tracks in the *xy*- and *xz*-planes. As indicated by the colorbar, different dot colors correspond to different times. Supplementary Movie 4 shows the time-lapse QPMs and the complete 3D tracking of the three nuclei under test.

Of course, the movements of the nuclei within their cells appear to be affected by both translations and rotations. A full analysis of typical nuclear movements is reported in Fig. 3d, e and shown in Supplementary Movie 4. In particular, in Fig. 3d we plot the temporal trend of the observed area, *A*_*N*_, of each nucleus. The changes in the projected areas highlight a rotation around an axis that lies in the *xy*-plane. Instead, in Fig. 3e we plot the temporal trend of the orientation of each nucleus with respect to the *x*-axis, which underlines a rotation around the optical *z-*axis. Vertical lines in Fig. 3b, d, e indicate the plasmolysis event for the three cells under test.

**Fig. 4:**
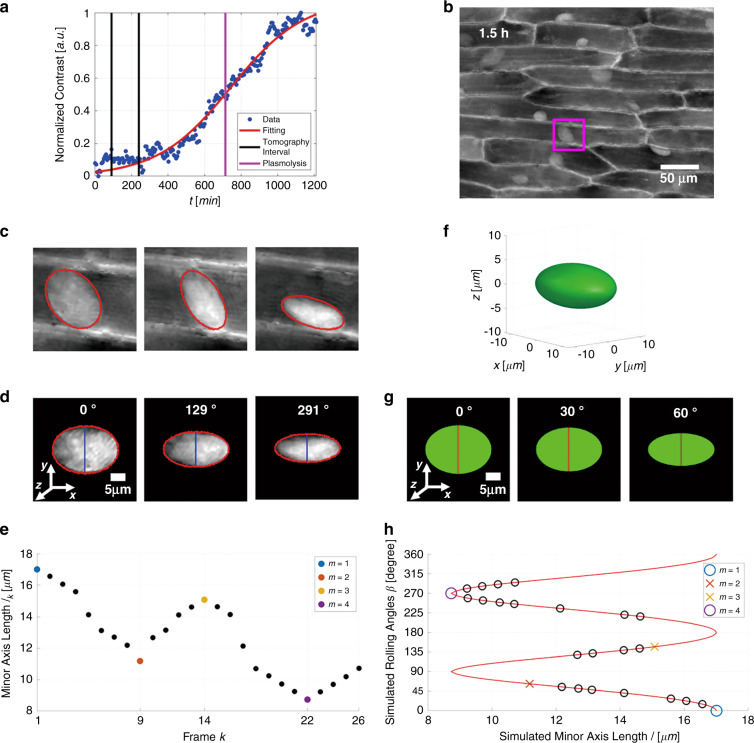
(Supplementary Movie 5) Rolling angle recovery method (see also “Materials and methods”). **a** Global image contrast normalized between 0 and 1, with overlapped in red the logistic fitting. The vertical black lines delimit the interval of frames used to perform the tomographic reconstruction of the analyzed nucleus (from 1.5 to 4 h), while the vertical violet line corresponds to the plasmolysis of the surrounding cell (12 h). **b** QPM at 1.5 h with highlighted in violet the ROI containing the analyzed nucleus (36.6 × 36.6 μm). **c** ROIs taken from three QPMs, with overlapped in red the contours of the elliptic binary masks. **d** Segmented QPMs in (**c**), centered and rotated to align the minor axes (in blue) along the *y*-direction of the reference system. At the top of each image, the corresponding recovered rolling angle is reported. The scale bar is 5 μm. **e** Minor axes’ lengths *l*_*k*_ of the elliptic binary masks of the same nucleus in the various frames *k*. The four colored dots are the extreme points *m* of the curve. **f** Silhouette of the nucleus simulated as an ellipsoid, according to the shape and the size of its segmented phase maps. **g** Three projections of the ellipsoid in (**f**) in the *xy*-plane, obtained by rotating it around the *x*-direction at the angles reported at the top of each image and by summing along the *z*-direction. Red lines are their minor axes. The scale bar is 5 μm. **h** Minor axes’ lengths *l* of the projections of the numerical ellipsoid in (**f**) versus the simulated rolling angles *β* (red line). Circles and crosses are the recovered rolling angles *θ*_*k*_ corresponding to the minor axes’ lengths *l*_*k*_ in (**e**). For the sake of clarity, only one of the two possible determinations of the estimated angles for *m* = 2 and *m* = 3 is shown in (**h**) by colored crossed. Due to the ambiguity, such angles are not considered here for PCT

Note that the roto-translations of the cells’ nuclei can be already observed at the beginning of the dehydration process. As shown in Fig. 1c, although plasmolysis occurs only when the rate of aqueous loss is maximum, the increase of contrast and in turn, the decrease of turgor pressure is continuous within the observation window. Therefore, even when the cell membrane has not detached from the cell wall, the reduction of the turgor pressure causes internal cell flows and a non-negligible relaxation of the intermediate filaments. As a consequence, the nucleus can start moving within the cell. This is a very important property. Indeed, in the following, we describe how to exploit this induced rotation process to perform phase-contrast tomography of the nucleus, in order to estimate the associated refractive index distribution and 3D morphometry at the sub-nuclear level. The temporal interval in which we use QPMs for the tomographic reconstruction of the nuclei is highlighted by two black vertical lines in Fig. 4a, overlapped to the global normalized contrast. It goes from 1.5 to 4 h, i.e., much before the plasmolysis (vertical violet line at 12 h). This means that, to use the tomographic tool in order to perform a 3D analysis of the nucleus of a plant cell, plasmolysis is not a required event, and one can operate in a time window of total reversibility for the cell. Therefore, in principle, one could induce a controlled dehydration, make the tomographic reconstruction of the nucleus when the cell just starts to become flaccid, and then bring it back to the initial turgid state by hydration without any damage to the sample.

### Rolling angle tracking

Figure 4b shows the first QPM of the sequence we use for tomography, which corresponds to the first black line in Fig. 4a. A violet box indicates the region of interest (ROI) containing the analyzed nucleus. For each frame of the sequence within the black lines in Fig. 4a, an ellipse, having axes equal to the major and minor orthogonal sizes of the nucleus in that frame, is used for image segmentation. Figure 4c shows zoom-in details of the ROI, extracted from three QPMs containing the same nucleus that rotates during the observation interval, along with the corresponding ellipses. The nucleus is extracted from each image, centered with respect to its centroid, and rotated in the image plane *xy* to align the major axis to the *x*-direction, as reported in Fig. 4d. In this way, the nucleus rotation can be detected from the variation of the minor axes’ lengths, highlighted by the blue lines in Fig. 4d, while the major axis keeps fixed. Figure 4e reports the minor axes’ lengths in each frame of the sequence, showing a sinusoidal trend and providing four local extremes *m* = {1, 2, 3, 4} (colored dots). According to the shape and the variation of the segmented phase maps in Fig. 4c, d, the rotation of the nucleus appears as the rotation of a plate around an axis parallel to its major face. Therefore, we assume that the nucleus makes a rotation of 90° between two successive extremes. We exploit this behavior to recover the unknown rolling angles employing the method proposed in ref. ^38^, based on the 3D ellipsoid fitting. The procedure developed to estimate the rotation angles is detailed in the “Materials and methods” section. The rotation angles, *θ*_*k*_, estimated using the angle tracking algorithm are indicated in Fig. 4h by circles and crosses. As shown in Fig. 4h, according to the choice of the axes of the simulated ellipsoid, the difference between the recovered rolling angles corresponding to the global extreme points is an entire multiple of 90°. Therefore, we can estimate that between the global extremes (*m* = 1 and *m* = 4) a 270° rotation occurs (Fig. 4h). Instead, *m* = 2 and *m* = 3 cannot be considered the points in which the rotations at 90° and 180° occur, since they are only local extrema, not corresponding to a global minimum or maximum. For this reason, after ordering the fitted angles, we exclude the images corresponding to angles in the local extreme points since they are indeterminate. In Fig. 4h, the excluded data are represented by colored crosses. The angle tracking algorithm described above and in the “Materials and methods” section is summarized in Supplementary Movie 5. The proposed technique allows recovering the unknown rolling angles of the plant cells’ nuclei without having any prior information.

It requires the recognition of at least one minimum−maximum or maximum−minimum transition in the curve of minor axes’ lengths because we use these extreme values to simulate the rotation of an ellipsoid with the same size of the segmented nucleus. Moreover, the nucleus must rotate around a single rotation axis that lies in the image plane. In the proposed example, this property is satisfied because the length of major axes is always the same, i.e., *L* = 23.44 μm, and the nucleus has never a rigorously circular projection in the various frames, as confirmed by the comparison between the major axis length and the values in Fig. 4e. In addition to the rolling angles, by using this method we obtain the segmented and centered phase maps, aligned to have the rotation around the *x*-direction, as shown in Fig. 4d. Combining angle tracking and 3D centroid tracking, we can follow the 3D roto-translation of the nuclei within the FoV fulfilling the abovementioned conditions. In the cases in which a sufficient number of views of the nucleus is available, the set of centered phase maps and the associated angles constitute the input of the filtered back-projection algorithm^47^ to reconstruct the 3D tomogram of a plant cell nucleus.

### Phase-contrast tomography of the nucleus

**Fig. 5:**
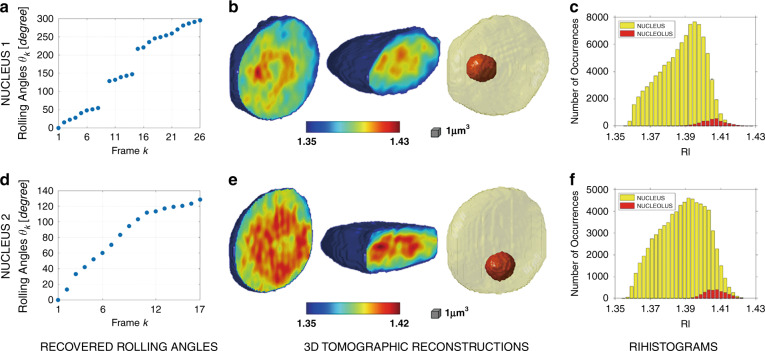
(Supplementary Movie 6) 3D tomographic reconstructions of two plant cells’ nuclei, captured under green laser light. **a, d** Rolling angles recovered by the minor axes’ lengths of the elliptic binary masks used to segment QPMs. **b, e** Central slices taken from the 3D reconstructed tomograms along two different directions (first two images) and iso-levels representation with the nucleolus highlighted in red (third image). **c, f** Histograms of the RI distribution of the nucleus (yellow) and the nucleolus (red)

In Fig. 5 we report the results of the 3D tomographic pipeline for the two analyzed nuclei. The first row reports the reconstruction of the nucleus in Fig. 4. In Fig. 5a we show the rolling angles recovered by using the method described above. To reconstruct this nucleus, we used 24 of the 26 recorded frames, since we excluded two frames corresponding to the undetermined local extreme points, as previously discussed. For this reason, the plot in Fig. 5a exhibits two discontinuities. The two leftmost images in Fig. 5b show two central slices of the 3D reconstructed nucleus, cut along two orthogonal directions. The tomographic technique allows to obtain the 3D spatial distribution of refractive index (RI). In the leftmost slice in Fig. 5b, it is clear the presence of a localized region at the highest RI values, which can be identified as the nucleolus. Therefore, we segment the nucleus in nucleoplasm and nucleolus by finding the corresponding interval of RI values. The third image in Fig. 5b is an iso-levels representation of the nucleus, in which the nucleolus is highlighted in red. Finally, in Fig. 5c the histograms of the RI values of the nucleus and nucleolus are reported. It is clear that, the segmented object (nucleolus) is well defined and separated from the surrounding medium (nucleoplasm) because of the unimodality of its RI distribution. In order to confirm the correct identification of the segmented object as the nucleolus, we compared its shape and relative size (with respect to the whole nuclear size) with the values that can be inferred from the images reported in literature, recorded by a confocal microscope^48^, a transmission electron microscope^49,50^, and an atomic force microscope^51^. Noteworthy, we found a very good agreement between the result obtained using the proposed QPI approach and the other gold standard methods. However, one of the main advantages of PCT with respect to more conventional imaging techniques is the possibility to measure quantitative 3D morphological features and RI statistics of the sample. In this case, dehydration induces phase-contrast enhancement and rotation, allowing such quantification at the sub-nuclear level. In the first column of Table 1, some of these quantities are reported for the example of Fig. 5a−c. In particular, an important biological parameter is the dry mass *m*_*d*_, i.e., the mass of the cell without the aqueous content^52,53^. It can be calculated from the reconstructed tomogram^22,52–54^ as follows:

1 $${{{\boldsymbol{m}}}}_{{{\boldsymbol{d}}}} = \frac{{\left( {{{{\bar{\boldsymbol n}}}} - {{{\boldsymbol{n}}}}_{{{\boldsymbol{w}}}}} \right){{{\boldsymbol{V}}}}}}{\boldsymbol\alpha }$$

where $\bar n$ is the mean value of RI, *n*_*w*_ = 1.334 is the RI of water, *V* is the volume and *α* is the refraction increment^55^, reported for proteins and DNA to be *α* ≈ 0.2 ml g^−1^. In order to obtain a reliable tomogram, the maximum illumination angle should be at least 180°. However, sometimes the sample does not complete a 180° rotation in the recorded holographic sequence.

**Table 1:** Quantitative measurements in 3D reconstructed tomograms of plant cells nuclei.

	MO = 25× *λ* = 532 nm		MO = 20× *λ* = 632.8 nm				Mean ± std (*)
	Cell 1	Cell 2	Cell 3	Cell 4	Cell 5	Cell 6	
Nuclear volume [μm^3^]	1377.42	1085.82	2664.99	1954.09	3275.01	3102.74	2066.85 ± 844.14
Nuclear equivalent radius [μm]	6.90	6.38	8.6	7.76	9.21	9.05	7.75 ± 1.14
Nuclear 1st principal axis [μm]	19.61	19.22	25.22	20.63	24.73	23.74	21.58 ± 3.87
Nuclear 2nd principal axis [μm]	13.89	14.97	14.35	13.79	16.00	15.50	14.50 ± 1.98
Nuclear 3rd principal axis [μm]	6.98	5.26	10.14	9.51	11.41	11.58	8.84 ± 2.22
Nuclear average RI	1.39	1.39	1.38	1.38	1.38	1.38	1.38 ± 0.01
Nuclear standard deviation RI	0.01	0.01	0.01	0.01	0.01	0.01	0.01 ± 0.00
Nuclear minimum RI	1.35	1.35	1.36	1.35	1.35	1.35	1.35 ± 0.01
Nuclear maximum RI	1.43	1.42	1.42	1.42	1.41	1.40	1.42 ± 0.01
Nuclear dry mass [pg]	370.51	295.25	599.47	482.00	722.34	660.85	498.90 ± 180.80
Nucleolar volume [μm^3^]	50.25	40.12	82.69	55.63	101.97	106.88	69.62 ± 27.24
Nucleolar equivalent radius [μm]	2.29	2.12	2.70	2.37	2.90	2.94	2.51 ± 0.35
Nucleolar 1st principal axis [μm]	4.95	4.53	5.32	4.93	5.67	6.18	5.20 ± 0.67
Nucleolar 2nd principal axis [μm]	3.96	3.77	5.14	3.99	5.13	4.93	4.40 ± 0.62
Nucleolar 3rd principal axis [μm]	3.56	3.25	4.21	3.90	4.85	4.82	4.01 ± 0.69
Nucleolar average RI	1.41	1.41	1.40	1.39	1.39	1.38	1.40 ± 0.01
Nucleolar standard deviation RI	0.01	0.01	0.01	0.01	0.01	0.01	0.01 ± 0.00
Nucleolar minimum RI	1.37	1.38	1.38	1.37	1.37	1.35	1.37 ± 0.01
Nucleolar maximum RI	1.43	1.42	1.42	1.42	1.41	1.40	1.42 ± 0.01
Nucleolar dry mass [pg]	17.95	14.58	26.77	16.27	31.07	26.03	21.97 ± 8.12
Nucleolar-nuclear volume ratio [%]	3.65	3.70	3.10	2.85	3.11	3.44	3.41 ± 0.31

The values reported in the column marked with (*) are estimated over 44 nuclei.

An example is reported in Fig. 4d−f. To reconstruct this nucleus, we used all the 17 recorded frames, recovering a maximum rolling angle of about 130°. In fact, if the maximum rolling angle is lower than 180°, the curve of minor axes’ lengths contains only global extreme points, so the final check about the local extreme points described above is useless. For this reason, unlike Fig. 5a, in Fig. 5d no discontinuity can be observed. Despite the angular discontinuities, the tomogram in Fig. 5b has a higher quality because the maximum recovered rolling angle is about 300°, meaning that more information is available for the reconstruction. However, although the reconstruction quality in Fig. 5d−f is lower due to the limited maximum rolling angle, we measured RI statistical features close to those of the first nucleus, as reported in the second column of Table 1, and, more importantly, we were still able to segment the nucleolus from the surrounding nucleoplasm. Finally, we underline that this second nucleus was extracted from another holographic sequence, but even in this case, the tomographic window was placed in time much before the critical event, i.e., the plasmolysis. Supplementary Movie 6 summarizes the results reported in Fig. 5 and offers a complete view of the 3D tomograms, showing all the reconstructed slices and the iso-levels representation of nucleolus and nucleoplasm.

Among the several quantitative parameters reported in Table 1, it is worth remarking the nuclear average RIs, i.e., 1.39 in both cases. In addition, we also measured the average RI of the cytoplasm (see “Materials and methods”) using a separate DH analysis, obtaining a value of 1.368 that is consistent with the measurements reported in literature^56^.

**Fig. 6:**
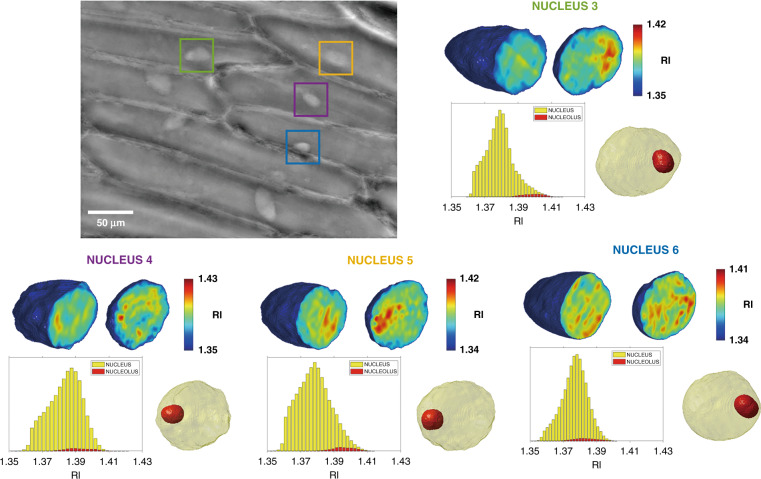
(Supplementary Movie 7) Tomographic reconstructions of four plant cells’ nuclei captured under red laser light. For each nucleus, marked with colored boxes in the QPM, the central slices taken from the 3D reconstructed tomograms are shown along two different directions. The corresponding histograms of the RI distribution of the nucleus (yellow) and the nucleolus (red) are reported, along with the iso-levels representation with the nucleolus highlighted in red

In order to assess the reproducibility of the proposed method, two additional experimental configurations have been employed, respectively using a 20× and a 10× microscope objective (MO). The latter was adopted at the scope of enlarging the FoV significantly in order to observe the dehydration process over a larger number of cells. Details of these holographic setup configurations are reported in the Supplementary Information section. In all the recorded time-lapse sequences, we observed roto-translations of the plant cells nuclei upon dehydration starts. In total, by using the three experimental configurations, we calculated 44 tomograms, identified the corresponding nucleoli, and measured all their relevant quantitative information. The rightmost column in Table 1 reports the average values of the estimated parameters, measured over all the 44 nuclei, along with the corresponding standard deviations. Figure 6 shows one of the captured QPMs obtained from the configuration employing the 20× MO, where four nuclei are marked with colored boxes (the corresponding quantitative parameters are reported in Table 1). For each of them, we show the central slices taken from the 3D reconstructed tomograms along two different directions. Besides, we show the corresponding histograms of the RI distribution of the nucleus (yellow) and the nucleolus (red), along with the iso-levels representation with the nucleolus highlighted in red. Supplementary Movie 7 summarizes the results shown in Fig. 6 and shows all the tomographic slices for the four reported nuclei. Similarly, Fig. S3 shows the 3D tomograms, RI histograms, and the isolevel representations of the other nine nuclei, where the corresponding nucleoli are shown in red color (the corresponding quantitative parameters are reported in Tables S1, S2). As mentioned before, the proposed method discards the nuclei experiencing a rotation with maximum rolling angle smaller than 90°, since in those cases the angle tracking algorithm would not furnish reliable results. In this sense, in Fig. 6 the nuclei without colored boxes cannot be reconstructed although they show roto-translations. Moreover, in order to provide a further indication of the number of nuclei that are expected to experience the required rolling for PCT, Fig. S4 and Supplementary Movie 8 show one of the QPMs obtained with the 20× MO and one of the QPMs obtained with the 10× MO, which allows accessing a wider FoV. In Fig. S4, we marked with a red box the nuclei experiencing rotations with maximum angles larger than 90°, and report two examples of tomographic reconstructions (denoted as nucleus 16 and nucleus 17 in Tables S1, S2). It is worth pointing out that all the observed nuclei have exhibited a roto-translation profile as a result of induced dehydration. In the QPMs of Fig. S4, about 65% of them are useful for PCT. Besides, the quantitative measures obtained with three different holographic setups are consistent with each other, which suggests the correctness and robustness of the reported results. The proof of concept reported in this paper shows the potential of the proposed technique. This could be exploited as a quantitative, label-free, non-invasive method to investigate further the nucleus and its composition. Differently from the method shown in ref. ^55^, the proposed approach does not need to break the cell to access the nucleus alone for measuring its refractive index. Indeed, we can study the nucleus composition while this is in its natural cell environment, thus operating in fully non-invasive conditions. Moreover, quantitative measurements about the nucleolar morphometric features and its dry mass are a precious source of information, since the nucleolus contains most of the genetic material of the cell.

## Discussion

Measuring physical and optical properties of intracellular structures is a highly pursued goal. In particular, quantifying the refractive index and dry mass of cell nucleus, and determining the nucleus/cytoplasm biovolume ratio within a cell during its life cycle are very debated topics. Recently, some evidence has been provided that the nucleus refractive index is lower than the surrounding cytoplasm for some specific cell lines^55^, calling into question the conventional common knowledge on this issue. Besides, providing such quantification at sub-nuclear level using a reagent-free method is a fascinating pathway. Acoustically-generated controlled microbubbles in a lab-on-a-chip device have been used to create micro-vortexes and induce rotation of pollen grains to perform 3D fluorescence microscopy of these cells^57^. However, such microfluidic manipulation using forces that are external to the cell cannot control the intracellular elements such as the nucleus, and the method is not quantitative. In this work, we have tuned the lab humidity and temperature conditions to induce a dehydration process in onion epidermal cells. We have observed them through holographic time-lapse recordings during a previously determined optimal time window within which the process is reversible, i.e., before plasmolysis starts. Dehydration increases the phase-contrast between the cell nucleus, cytoskeleton, and the surrounding cytoplasm, thus enhancing our ability to observe tiny subcellular structures through a limited sensitivity phase-contrast process. Thus, we observed cytoplasm circulation, intracellular transport phenomena, along with nucleus 3D movements and rotations. Such movements should be traced back to the changes in the hydrodynamic equilibrium state and the intrinsic cell reorganization upon dehydration starts, in agreement with the simplified model and the corresponding 3D finite-element numerical simulation. Combining centroid and angle tracking proved to be effective in retrieving tomographic maps of the cells’ nuclei. Studying the tomograms and the refractive index statistics allowed us isolating the nucleolus from the nucleoplasm in the 3D tomograms under analysis, and to measure its refractive index, 3D morphometry, biovolume, and dry mass. To the best of our knowledge, this is the first label-free refractive index and biovolume quantification at sub-nuclear level, made possible here by the “virtual” increment in system sensitivity that allowed us to accurately 3D track and refocus the nuclei in each frame of the time-lapse sequence. The recovered morphological information and the size ratio between the nucleolus and nucleoplasm are in good agreement with values reported in literature referring to more conventional invasive techniques^48–51^.

We believe this approach is very promising due to its non-invasiveness, since dehydration and the corresponding variation to the hydrodynamic conditions within the cytoplasm is an intrinsically natural process each cell is subject to, and that does not provoke any damage or irreversible effect to the cell. In nature, plants continuously experience dehydration-hydration cycles during their lifetime. Thus, before the plasmolysis event, dehydration is naturally non-destructive. Having exploited, for all the PCT analysis, an observation window placed in time much before the plasmolysis event (see also Fig. 4a) is an encouraging result that corroborates this idea. In the case of onion epidermal cells, we found that the cell nucleus has higher refractive index than the cytoplasm, and that the nucleolus has higher refractive index than the nucleoplasm. Isolating the nucleolus through PCT and thus measuring its quantitative features (see Table 1) could be pivotal in future perspectives to infer information about the content of genetic material in some cell lines. The natural dehydration process is found to be slow enough to ensure that the nuclei are not subject to compression during rotation. As an evidence of this argument, the measure of the nuclear projected area we observed in all the cases is oscillating (see Fig. 4e), therefore it is unlikely that the nuclei are subject to any deformation during the rolling process.

Possible developments of the method will address how to reinforce analysis tools, relying on machine learning to better analyze the tomograms and extract meaningful 3D features from the main sub-cellular and sub-nuclear components. It is worth pointing out how DH imaging allowed us to analyze and model the whole dehydration process towards plasmolysis. In particular, from the sole analysis of the normalized contrast of Fig. 1c we were able to deduce useful information on the reduction of turgor pressure and how this process develops in time, which is well fitted by a logistic curve. Moreover, we found that the derivative of the fitting curve has a peak in correspondence with the breaking event of the process, i.e., the convex plasmolysis with the detachment of the cell membrane from the cell wall. Even more important, we demonstrated that, in order to reach the aforementioned tomographic results, plasmolysis is not a required event, and one can operate in a time window of total reversibility for the cell. Therefore, in principle one could induce a controlled dehydration, make the tomographic reconstruction of the nucleus when the cell just starts to become flaccid, and then bring it back to the initial turgid state by hydration. The presented results are a proof of concept of the possibility to exploit a natural biological process as dehydration in a functional way, i.e., as a sort tool that allows 3D tomographic imaging of nuclei in plant cells using a conventional setup. Thanks to this fascinating possibility, we have achieved nucleus PCT, and we have clearly identified and quantitatively characterized the nucleolus. Although this has been applied here solely to epidermal onion cells, we expect it can be easily extended to all similar plant cells. In these cases, the optimal observation time window in which rotation occurs in a reversible modality is expected to change and should be estimated first. The rolling and shift behavior of nuclei belonging to different cell lines, and the optimal external conditions to induce such behavior will be further investigated in future studies. However, it is worth pointing out here that most of the cells we analyzed have shown roto-translations of their nuclei, although the 3D positions and angular tracking profile may change from cell to cell of the same line. However, in this method, the set of positions and view angles is estimated directly from experimental data, i.e., not assumed or inferred from models, which makes this approach robust against such changes occurring from cell to cell of the same type or belonging to different lines. Compared to illumination scanning PCT methods, which require complicated optical setups for beam rotators^58^, our system is able to collect a whole range of viewing angles. Moreover, it is well-known that in sample rotating PCT scheme, reconstructed tomograms exhibit isotropic spatial resolution along the lateral and axial directions. Usually, rotary micro-capillary or optical tweezers are employed to rotate the sample^58^, instead, our approach completely avoids any rotation strategy because nuclei rotation is naturally induced by dehydration. Of course, our main drawback is the longer recording time needed to collect multiple views of nuclei due to the slowness of the dehydration process. In order to make the method faster, the process of water exchange could be helped further. In principle, providing an osmotic stress to the cells would force water to be drawn out of the cell membrane by osmosis. This would provoke a rapid change of the hydrodynamic balance within the cell and thus a sudden shock forcing the nucleus to move and rotate. On the other hand, a too rapid and not natural change of the cytoplasmic balance within the cell could provoke tiny deformations to the nucleus, thus affecting tomographic reconstructions. As an alternative solution, we could reduce the data acquisition time, and consequently collect limited angle views, but a more sophisticated reconstruction algorithm, able to preserve the tomography accuracy, is needed^59^. However, as pointed out in the section 2.4, our method is based on the calculation of nucleus orientations that requires the recognition of at least one minimum−maximum or maximum−minimum transition in the curve of minor axes’ lengths, i.e., a sufficient number of views must be collected. We believe future works should be devoted to investigate further these interesting possibilities, e.g., with the aim to identify an optimal sample buffer that trades-off convenient timing and non-invasiveness.

One more consideration is due. Since dehydration can be affected by processes of cell aging, estimating the optimal time window could be used as effective diagnostic tool to monitor plants’ health and its related biological processes. In fact, dehydration is a typical stressing factor for plants in situation of scarce water resources. The approach demonstrated here could improve the investigation in plant biology by a non-destructively controlled procedure and by means of a conventional label-free holographic microscope able to furnish 3D quantitative analysis at sub-nuclear level.

## Materials and methods

### Digital holography processing

**Fig. 7:**
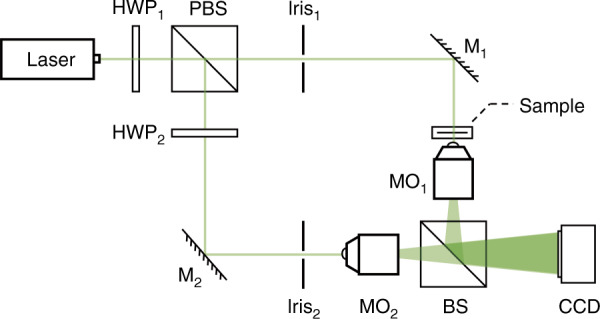
Experimental setup. *HWP1/HWP2* half wave plates at wavelength 532 nm. *PBS* polarizing beam splitter. *M1/M2* mirrors. *MO1/MO2* microscope objectives. *BS* beam splitter

Let *H* be the digital hologram captured in off-axis configuration (Fig. 7). This is the coherent superposition of the object beam, *O*, and the reference beam, *R*:

2 $${{{\boldsymbol{H}}}} = \left| {{{{\boldsymbol{R}}}} + {{{\boldsymbol{O}}}}} \right|^2 = \left| {{{\boldsymbol{R}}}} \right|^2 + \left| {{{\boldsymbol{O}}}} \right|^2 + {{{\boldsymbol{R}}}}^ \ast {{{\boldsymbol{O}}}} + {{{\boldsymbol{RO}}}}^ \ast$$

The angle between *O* and *R* allows decoupling the object image from the twin image and the zeroth-order term by trivially demodulating Eq. 2. In the case the object of interest is captured out of focus, numerical propagation of the demodulated hologram, *H*_*d*_, allows retrieving the object complex amplitude in the best focus plane, $C = P_{z_F}\left\{ {H_d;z_F} \right\}$, where *P*_*z*_ {…} is the propagation operator that solves the diffraction integral^60^. The best-focus distance, *z*_*F*_, maximizes a proper contrast metrics, namely the Tamura coefficient^37^ of the sample phase-contrast map *ψ*_*O*_ = *arg*{*C*}.

### Experimental setup

The off-axis DH setup is based on a Mach−Zehnder interferometer scheme with two coherent beams generated by a laser source of wavelength *λ* = 532 nm (Fig. 7). The laser source was a solid-state single longitudinal mode laser, MSL-III-532, from Changchun New Industries Optoelectronics Tech. CO., Ltd. The output power at the laser exit is 20 mW.

The laser spot is divided into two beams by a polarizer beam splitter prism (PBS), where the transmission beam is set as the object beam, and the reflected beam is set as a reference. One half-wave plate is placed in front of the PBS to adjust the splitting ratio of the two beams, and another half-wave plate is located behind of the PBS to adjust the polarization state of this beam. Living onion epidermal cells are prepared and loaded in between two pieces of glass slides and placed in the path of object beam, so that its amplitude and phase information can modulate the object wavefront. After passing through the beam splitter prism (BS), the object beam and reference beam with an angle between them are combined and generate an interference pattern, i.e., the off-axis digital hologram. Two identical GCO-2114 MOs with 25× magnification and 0.4 NA from Daheng New Epoch Technology, Inc. are used to provide a 26.43× overall imaging system magnification, measured using a USAF-1951 resolution target. A CCD camera with square pixels is employed to record the digital holograms. The CCD camera model is Ximea MD028MU-SY (1960 × 1460 pixels) with 4.54 μm pixel pitch. The area used for recording the holograms was 1280 × 960 pixels.

Thus, the pixel size in the image plane is *Δx* = *Δy* = 0.17 μm. In the optical setup, the two spherical waves from the object beam and reference beam have the same curvature. The effective spatial resolution in the image plane, measured using a USAF test target, is 0.24 μm. The holographic setup captures a *FoV* = 0.036 mm^2^. The setup is conceived to be minimally invasive for the sample layers, so that we kept the beam power on the sample plane lower than 21 µW (the measured average value was 19 µ*W*±10 %) during the entire experiment.

### Estimation of rotation angles

Let *l*_*k*_ be the minor axes’ lengths, with *k* *=* 1*,…,N* being the index of a generic frame and *N* the number of frames. Let *f*_*m*_ be the index of a generic frame containing an extreme point, with *m* *=* 1*,…,M*, where *M* is the number of extreme points. As shown in Fig. 4f, we generate a numerical ellipsoid, whose axes are defined as the major axis (along the *x*-direction), the maximum minor axis (along the *y*-direction), and the minimum minor axis (along the *z*-direction) of the ellipses used to segment the phase maps. Therefore, this ellipsoid approximates the silhouette of the rolling nucleus. We simulate the rotation of the ellipsoid from 0° to 360° around the *x*-direction. For each simulated rolling angle, *β*, we sum the values of the rotated ellipsoid along the *z*-direction to generate its projections, which have an elliptic shape in the *xy*-plane. Figure 4g shows three of these ellipses with the corresponding angles of projection, highlighting in red their minor axes. The so-obtained ellipses always have the same major axis, i.e., the major axis of the elliptic binary masks, while their minor axes’ lengths, *l*, take intermediate values from the maximum minor axis to the minimum minor axis measured in the elliptic binary masks. In Fig. 4h, the dependence of the simulated rolling angles *β* from the minor axes lengths *l* of the projected ellipses is plotted with a red line. The simulated ellipses in Fig. 4d are directly comparable with the elliptic binary masks in Fig. 4g. Therefore, for each measured minor axis length, *l*_*k*_, from the curve *β(l)* we extract the corresponding fitted rolling angles *β*_*k*_, with *k* *=* 1*,…,N*. The fitted angles, *β*_*k*_, are in the 0°−90 ° interval and, for *k* *=* 1*,…,N*, they follow a trend similar to the trend of the minor axes lengths, *l*_*k*_, in Fig. 4e. Therefore, we reorganize them in a crescent order by using the property discussed above, i.e., whenever the curve in Fig. 4e passes an extreme point *m*, the nucleus accumulates an additional 90° rotation. In this way, we recover the rolling angles, *θ*_*k*_, which we show as circles in Fig. 4g, also highlighting the extreme points of Fig. 4e. Moreover, at the top of the three segmented phase maps in Fig. 4d, we report the corresponding recovered rolling angles.

### Sample preparation

Samples of living onion epidermal cells were prepared from fresh onions with intact and undamaged fibrous roots. During sample preparation, the fourth layer of fleshy scale leaves from the center was used to prepare a single layer of onion epidermal cells. Then, the selected single-layer onion epidermal cells were loaded in between the two glass slides. During the whole experiment, the environment temperature in the laboratory was kept to 20 °C, and the humidity was maintained at 35%. Digital holograms of the onion epidermal cells were recorded under darkroom conditions.

### Measure of the cytoplasm refractive index

Fresh rooted onions were bought the morning of the experiment. The roots were immersed in water for 8 h. Twenty single-layer onion skins were peeled out, each with 2 × 5 cm area. Layers were stacked, gently crushed in a test tube, and then centrifugated to obtain the cell cytosol. A two-layer ashless filter paper was used to filter the cytosol out. The filtered cytosol was put in a sealed test tube and made stand for 1 h. The overall procedure has been repeated 15 times to obtain enough fluid for the measure. The obtained fluid was injected inside a 100 μm high Polymethylmethacrylate (PMMA) microfluidic channel and a DH was captured. Conventional DH processing was applied to obtain the phase of the liquid. A reference hologram was captured after filling a clean PMMA microfluidic channel with water. Since the channel height and its own refractive index, *n*_PMMA_=1.490, did not change between the measures, comparing the phase values provided the indirect estimation of the fluid refractive index, i.e., *n*_*C*_ = 1.368, which is in good agreement with the value reported in literature^56^.

## Supplementary information

Supplementary information - revised

1.Time lapse holographic phase-contrast observations of onion epidermal cells. Dehydration allows imaging the cell with enhanced phase-contrast

2.Finite element DNS of cell dehydration at increasing values of time before plasmolysis

3.Finite element DNS of cell dehydration at increasing values of time after plasmolysis

4.Observing nucleus movements induced by dehydration

5.Method to recover the rolling angles of the cells’ nuclei, to be exploited for 3D Phase Contrast Tomography (PCT)

6.3D tomographic reconstructions of two plant cells’ nuclei

7.3D tomographic reconstructions of four plant cells’ nuclei captured under red laser light

8.Time-lapse QPMs of onion epidermal cells captured under 20x and 10x magnification
