# Supplementary material for: Dehydration of plant cells shoves nuclei rotation allowing for 3D phase-contrast tomography
Source: Light Sci Appl. 2021 Sep 15;10:187. doi: 10.1038/s41377-021-00626-2 (PMC8443563; doi:10.1038/s41377-021-00626-2)
Supplement: Supplementary file 1 — Supplementary information - revised [file 41377_2021_626_MOESM1_ESM.docx]

**Supplementary information**

Dehydration of plant cells shoves nuclei rotation allowing for 3D phase‑contrast tomography

Zhe Wang,^1,2^ Vittorio Bianco,^2,3^ Daniele Pirone,^3,4^ Pasquale Memmolo,^2,3,*^ Massimiliano Maria Villone,^1,2^ Pier Luca Maffettone,^1,2^ Pietro Ferraro^2,3,**^

*^1^ Dipartimento di Ingegneria Chimica dei Materiali e della Produzione Industriale, Università degli Studi di Napoli "Federico II",* *Piazzale Tecchio 80, 80125 Napoli, Italy*

*^2^ NEAPoLIS, Numerical and Experimental Advanced Program on Liquids and Interface Systems, Joint Research Center CNR ‐ Università degli Studi di Napoli "Federico II", Napoli, Italy.*

*^3^ CNR-ISASI, Institute of Applied Sciences and Intelligent Systems “E. Caianiello”, Via Campi Flegrei 34, 80078 Pozzuoli, Napoli, Italy.*

*^4^ Dipartimento di Ingegneria Elettrica e delle Tecnologie dell’Informazione, Università degli Studi di Napoli “Federico II”, via Claudio 21, 80125 Napoli, Italy.*

^*^ [pasquale.memmolo@isasi.cnr.it](mailto:pasquale.memmolo@isasi.cnr.it) , ^**^ [pietro.ferraro@cnr.it](mailto:pietro.ferraro@cnr.it)

**Dehydration and plasmolysis in plant cells**

Unlike the animal cell, in the plant cell the cell membrane is surrounded by the cell wall. It contains the protoplast, that is composed by the cell membrane, the nucleus, the cytoplasm and its organelles. A typical organelle in plant cells is the vacuole, i.e. a roundish tank with an aqueous solution, called cell sap, surrounded by a membrane (tonoplast). Usually, in mature plant cells, it occupies between 80 % and 90 % of the cell volume. For this reason, the vacuole is responsible for cell turgor. Cell turgor, or turgor pressure, is a hydrostatic pressure exerted by the tonoplast that pushes the cell membrane against the cell wall. This pressure regulates the solidity level of the cell. The cell is flaccid when the turgor pressure is low and the cell is turgid when the turgor pressure is high. The turgor pressure depends on the aqueous content of the vacuole. Indeed, in turgid cells, the higher aqueous content exerts more pressure on the cell walls than in flaccid cells. Therefore, in a hypotonic environment, water enters the cell through the pores of its membrane. The cell swells by osmosis, but it is not subjected to lysis because the turgor pressure is counterbalanced by the resistance exerted by the cell wall. Instead, in hypertonic solutions, the cell becomes flaccid due to the decrease of the turgor pressure, because the vacuole loses a part of its aqueous content. If this state persists, the protoplast retracts further and then the cell membrane detaches from the cell wall. This phenomenon is called plasmolysis. The space created between the cell wall and the protoplast is called periplasmic space. According to the cell type and the cytoplasm viscosity, plasmolysis can be convex or concave.


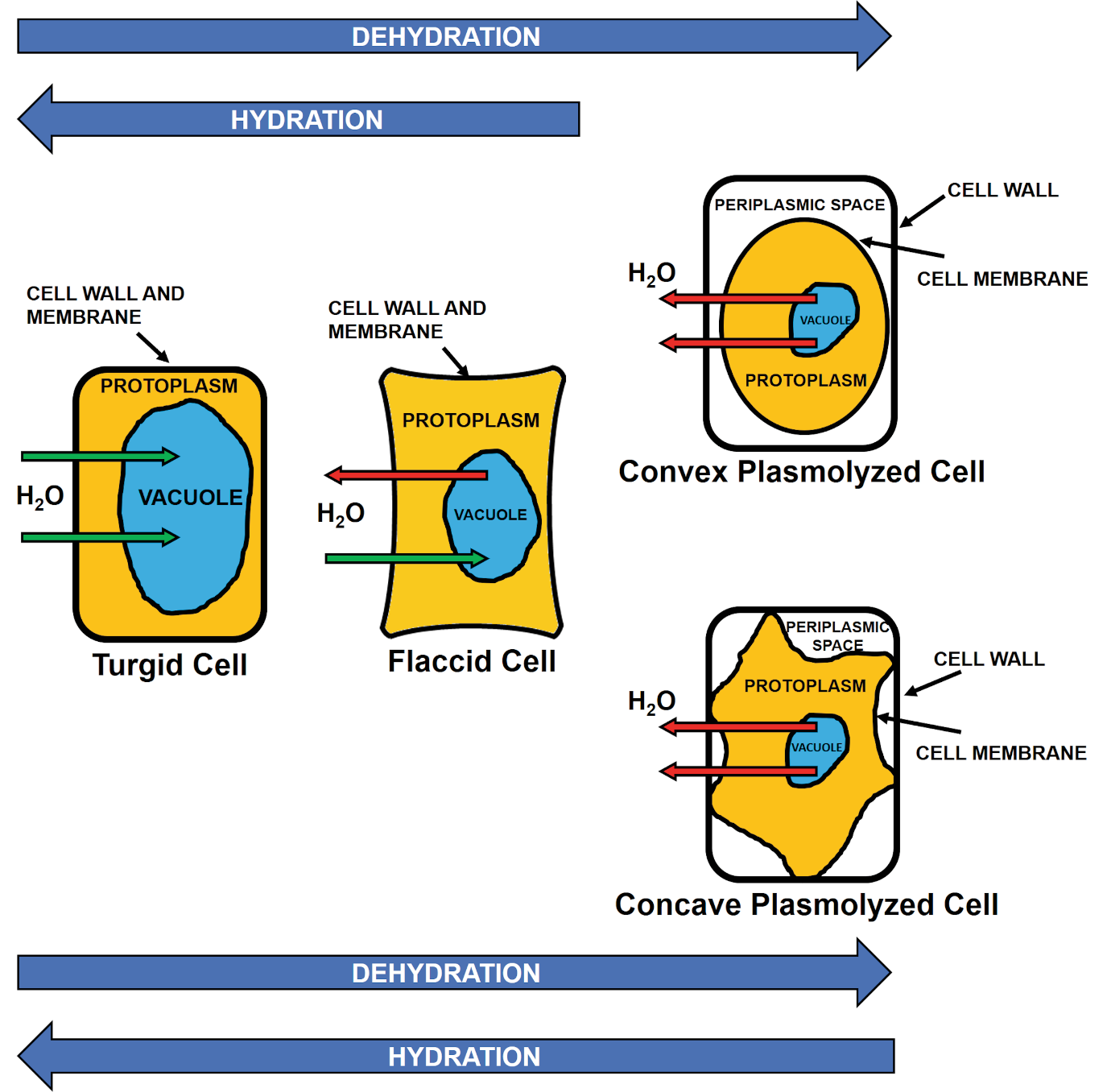


**Fig. S1. Sketch of the induced change in the cell turgor pressure due to the loss of cell aqueous content.** When cell is turgid, the vacuole occupies most of its volume. Due to the dehydration, the vacuole loses part of its aqueous content, thus the cell becomes flaccid. If this phenomenon persists, plasmolysis takes place. It can be concave or convex. Unlike the convex plasmolysis, the concave plasmolysis is reversible, so cell can recover its initial turgor through hydration (deplasmolysis).

In convex plasmolysis, the cytoplasm is rounded to form convex ends. In concave plasmolysis, the separation of the cytoplasm produces concave pockets.^33-36^ Unlike convex plasmolysis, concave plasmolysis is reversible because the initial turgor can be recovered by placing the cell in a hypotonic solution (deplasmolysis).

However, in both cases, the cell wall collapses if the plasmolysis persists, so causing the cell death. In Figure S1, the change of the cell status due to the variation of the aqueous content is sketched. In our experiments, cells are not dipped inside a hypertonic solution. Emptying of the vacuole is due to the evaporation of its cell sap thanks to the reduction of the environmental humidity down to the 35 % at constant 20 ° temperature. Tomographic observations are carried out in a time window that largely precedes convex plasmolysis (rotation of the nuclei are observed and exploited for PCT around 7 hours before the plasmolysis event for the cells we analyzed). This ensures we can operate in a time region of complete reversibility for the cells under test.

**Mathematical model for Direct Numerical Simulations**

As discussed in the Main Text, the fluid dynamics inside onion epidermal cells during dehydration is studied by Direct Numerical Simulations (DNS). For this purpose, a geometry retracing the shape and dimensions of cell 1 in Fig. 1 in the Main Text is considered (see Fig. S2). The computational domain is filled with water. Assuming that water is incompressible, and that gravity and inertia are negligible, the mass and momentum balance equations on the fluid in the Stokes regime are written, reading

$\left\{ \begin{aligned} \boldsymbol{\nabla}\boldsymbol{\cdot u=0} \\ \boldsymbol{-}\boldsymbol{\nabla}\boldsymbol{p+\eta}\boldsymbol{\nabla}^{\boldsymbol{2}}\boldsymbol{u=0} \end{aligned} \right.$ (S1)

with $\nabla$ the nabla operator, $u$ the fluid velocity field, $p$ the pressure field, and $\eta$ the fluid viscosity. In order to be solved numerically, the above-reported equations are supplied with boundary conditions. In the time-window of interest for tomography, the cell undergoes loss of turgor due to dehydration, but no plasmolysis occurs. In the perspective of the simplified model, this is obtained by imposing water evaporation through the top surface of the cell parallel to the *xy*-plane at a rate of 0.121 kg m^-2^ hr^-1^). Such indicative value is an estimate of water evaporation rate at room temperature in absence of air convective flow. In addition, since there is no plasmolysis, the no-slip/no-penetration condition is imposed on the “bottom” surface parallel to the *xy*-plane, whereas null velocity is imposed in the orthogonal direction on the lateral surfaces parallel to the *z*-direction. The equations describing the system are solved with the finite-element method on a Lagrangian unstructured tetrahedral computational grid by means of the commercial software COMSOL Multiphysics™ V5.5 (ref. S1). Due to water evaporation, the cell deflates in the *z*-direction as shown in Fig. 2a in the Main Text and in Supplementary Movie 2, thus triggering the onset of the flow field represented through the red streamlines in both the above-mentioned figure and video.

For completeness, the second phase, successive to the plasmolysis event, is simulated by imposing water evaporation on the cell lateral sides too. The results of such simulation are reported in Supplementary Movie 3 and Fig. 2b, showing both the cell shape evolution and the fluid streamlines inside it. It is apparent that fluid dynamic conditions compatible with the experimentally observed movements of the nucleus hold inside the cell also during the plasmolysis.


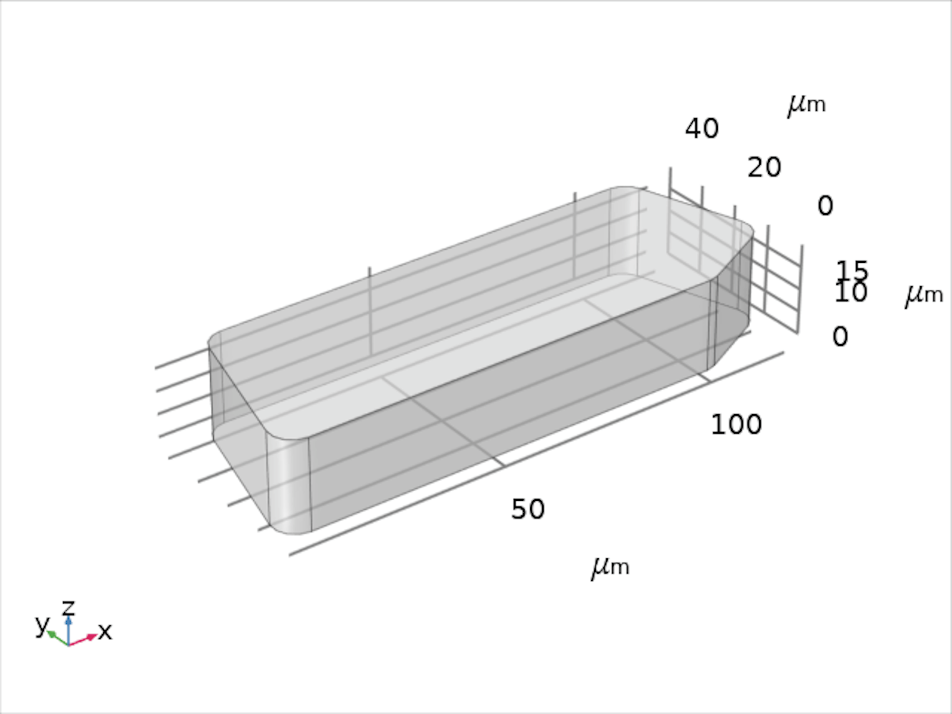


**Fig. S2. Geometry of the 3D domain for finite-element DNS of cell dehydration.**

**Experimental configurations adopting 20× and 10× microscope objectives**

The second and third setup configurations make use of a Thorlabs HNL150LB laser emitting at 632.8 nm. The CCD camera model was a Ximea MD028MU-SY, 4.54 μm pixel pitch, 1960×1460 active area. First, a 20× microscope objective (GCO-2107, NA=0.4) was used, providing a 24.95× effective magnification of the sample and a 352.3 μm × 265.0 μm maximum FoV. Then, this has been replaced by a 10× microscope objective (GCO-2102, *NA*=0.25), providing a 13.59× effective magnification in the image plane, and a 646.6 μm $\times$ 486.3 μm maximum FoV. Both objectives are from Daheng New Epoch Technology, Inc. Nuclei denoted with 1,2,7,8 have been captured using the setup employing the 25× objective (green laser light), described in the main text. Nuclei denoted with 3-6,9-13,16 have been captured using the setup employing a 20× objective (red laser light). Nuclei denoted with 14,15,17 have been captured using the setup employing a 10× objective (red laser light).


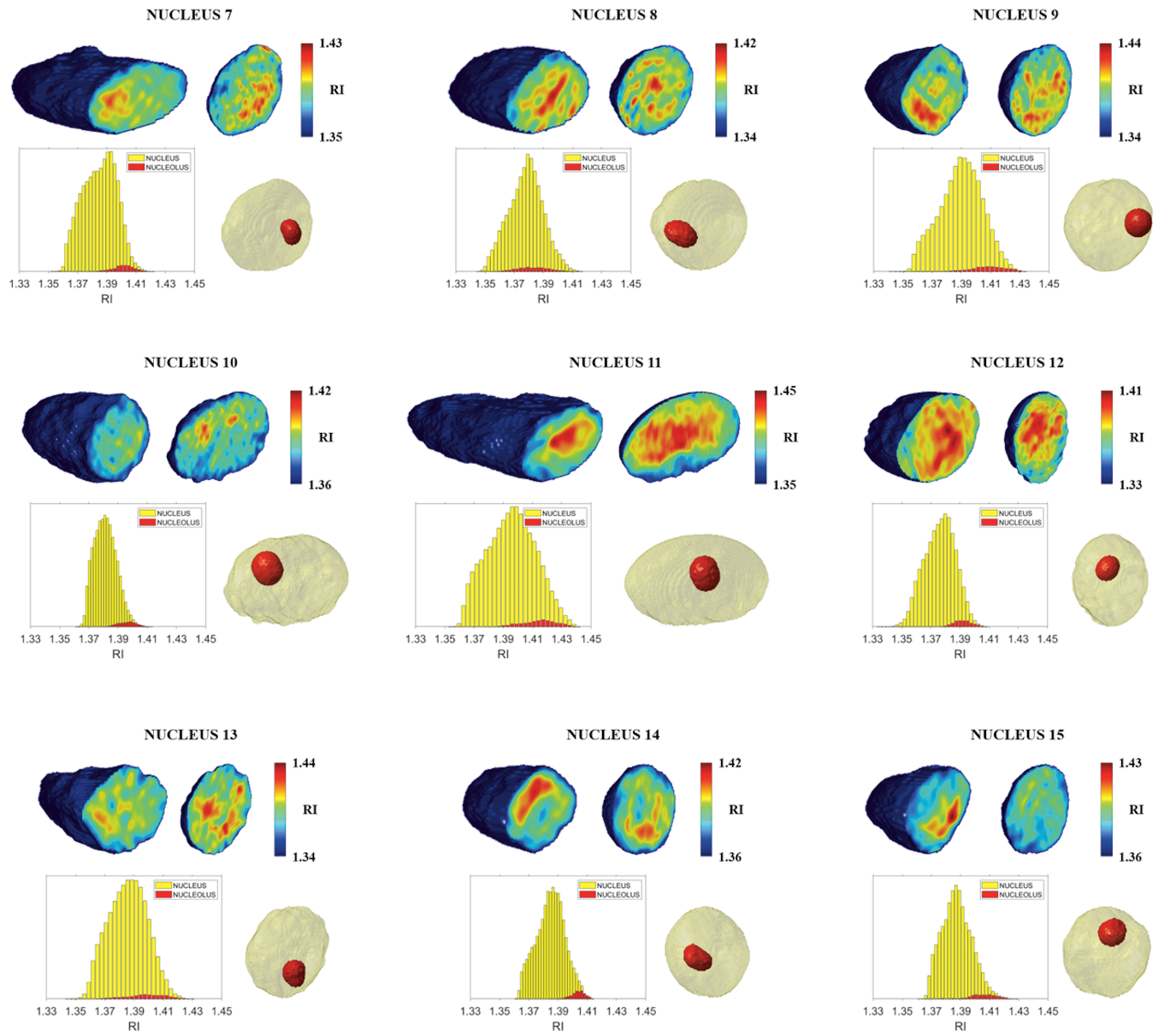


**Fig. S3 3D tomograms of nuclei denoted with numbers 7-15.** For each nucleus, the central slices taken from the 3D reconstructed tomograms are shown along two different directions. The corresponding histograms of the RI distribution of the nucleus (yellow) and the nucleolus (red) are reported, along with the iso-levels representation with the nucleolus highlighted in red.


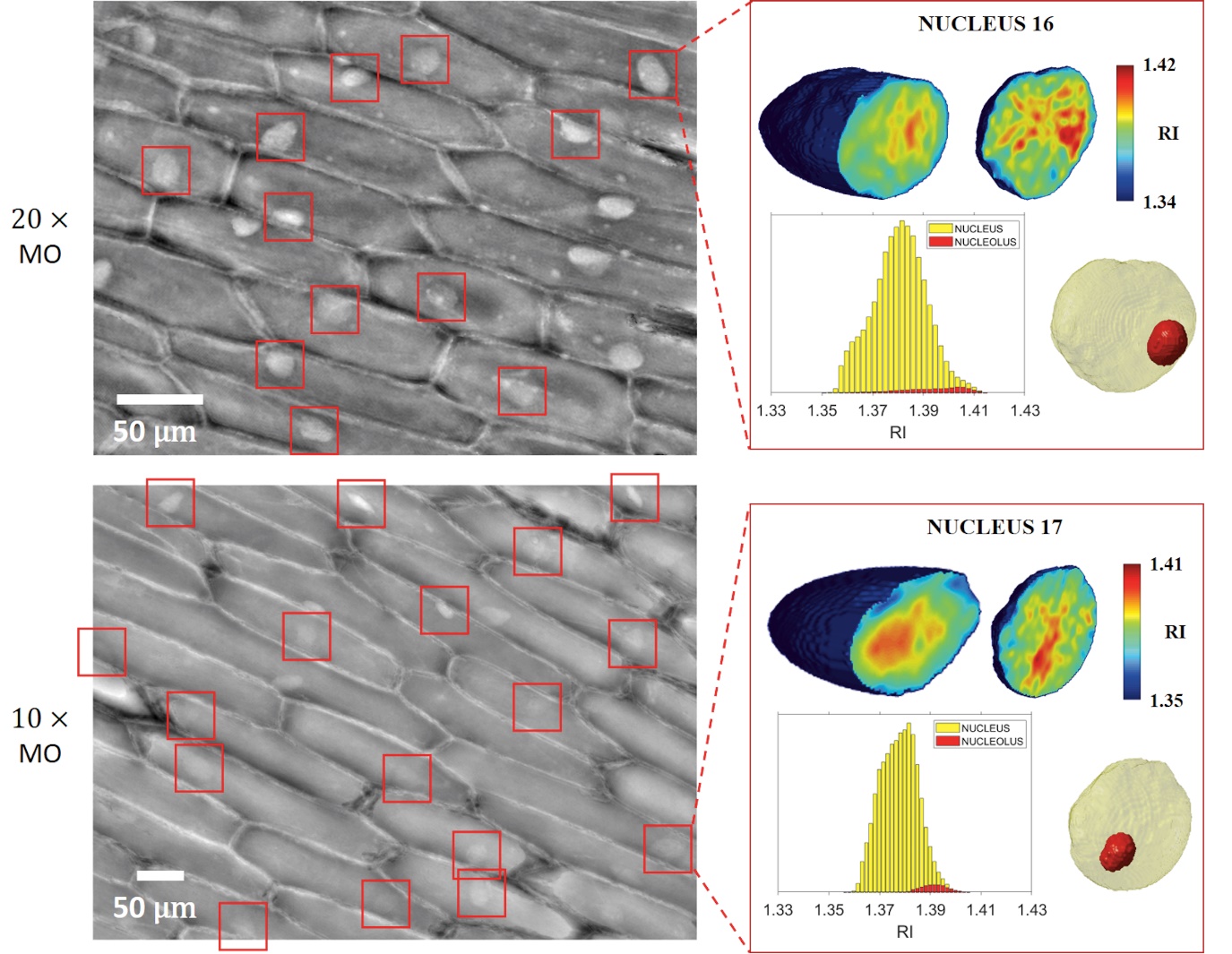


**Figure S4** (Supplementary Movie 8) **Full FoV QPMs captured using the (top) 20× and (bottom) 10× MOs.** The nuclei marked with the red box are found to experience rotation with maximum rolling angle higher than 90 °, thus being useful for PCT. Examples of PCTs are reported on the right, showing the nuclei marked with numbers 16 and 17 in Tables S1,S2.

Table S1. Quantitative measurements in 3D reconstructed tomograms of plant cells nuclei.

|  | *MO* = 25×  *λ* = 532 nm | | *MO* = 10×  *λ* = 632.8 nm | | |
| --- | --- | --- | --- | --- | --- |
|  | cell 7 | cell 8 | cell 14 | cell 15 | cell 17 |
| Nuclear Volume [μm^3^] | 1679.09 | 779.58 | 1854.94 | 2233.23 | 3581.07 |
| Nuclear Equivalent Radius [μm] | 7.37 | 5.71 | 7.62 | 8.11 | 9.49 |
| Nuclear 1^st^ Principal Axis [μm] | 23.98 | 16.62 | 18.52 | 19.09 | 26.15 |
| Nuclear 2^nd^ Principal Axis [μm] | 15.77 | 10.92 | 14.27 | 15.02 | 19.04 |
| Nuclear 3^rd^ Principal Axis [μm] | 6.13 | 5.96 | 9.68 | 10.83 | 9.89 |
| Nuclear Average RI | 1.38 | 1.38 | 1.38 | 1.39 | 1.38 |
| Nuclear Standard Deviation RI | 0.01 | 0.01 | 0.01 | 0.01 | 0.01 |
| Nuclear Minimum RI | 1.35 | 1.34 | 1.36 | 1.36 | 1.36 |
| Nuclear Maximum RI | 1.42 | 1.42 | 1.41 | 1.43 | 1.41 |
| Nuclear Dry Mass [pg] | 427.51 | 171.58 | 472.64 | 598.20 | 784.05 |
| Nucleolar Volume [μm^3^] | 49.04 | 29.63 | 62.89 | 87.04 | 111.52 |
| Nucleolar Equivalent Radius [μm] | 2.27 | 1.92 | 2.47 | 2.75 | 2.99 |
| Nucleolar 1^st^ Principal Axis [μm] | 4.81 | 4.52 | 5.87 | 5.75 | 6.30 |
| Nucleolar 2^nd^ Principal Axis [μm] | 4.05 | 3.25 | 4.28 | 4.74 | 5.20 |
| Nucleolar 3^rd^ Principal Axis [μm] | 3.50 | 2.83 | 3.54 | 4.43 | 4.75 |
| Nucleolar Average RI | 1.40 | 1.38 | 1.40 | 1.40 | 1.39 |
| Nucleolar Standard Deviation RI | 0.01 | 0.01 | 0.01 | 0.01 | 0.01 |
| Nucleolar Minimum RI | 1.38 | 1.36 | 1.38 | 1.37 | 1.37 |
| Nucleolar Maximum RI | 1.42 | 1.42 | 1.41 | 1.43 | 1.41 |
| Nucleolar Dry Mass [pg] | 16.41 | 7.29 | 21.85 | 30.51 | 31.93 |
| Nucleolar-Nuclear Volume Ratio [%] | 2.92 | 3.80 | 3.39 | 3.90 | 3.11 |

Table S2. Quantitative measurements in 3D reconstructed tomograms of plant cells nuclei.

|  | *MO* = 20×  *λ* = 632.8 nm | | | | | |
| --- | --- | --- | --- | --- | --- | --- |
|  | cell 9 | cell 10 | cell 11 | cell 12 | cell 13 | cell 16 |
| Nuclear Volume [μm^3^] | 2641.12 | 2573.46 | 1236.59 | 1813.00 | 871.92 | 2412.37 |
| Nuclear Equivalent Radius [μm] | 8.57 | 8.50 | 6.66 | 7.56 | 5.93 | 8.32 |
| Nuclear 1^st^ Principal Axis [μm] | 19.90 | 27.10 | 27.27 | 16.53 | 15.24 | 23.24 |
| Nuclear 2^nd^ Principal Axis [μm] | 15.80 | 13.24 | 11.22 | 15.68 | 11.87 | 15.20 |
| Nuclear 3^rd^ Principal Axis [μm] | 11.58 | 9.91 | 5.62 | 9.62 | 6.68 | 9.41 |
| Nuclear Average RI | 1.39 | 1.38 | 1.40 | 1.38 | 1.39 | 1.38 |
| Nuclear Standard Deviation RI | 0.01 | 0.01 | 0.02 | 0.01 | 0.01 | 0.01 |
| Nuclear Minimum RI | 1.34 | 1.36 | 1.35 | 1.33 | 1.34 | 1.35 |
| Nuclear Maximum RI | 1.43 | 1.41 | 1.44 | 1.41 | 1.43 | 1.42 |
| Nuclear Dry Mass [pg] | 735.70 | 610.47 | 381.16 | 382.14 | 225.96 | 561.52 |
| Nucleolar Volume [μm^3^] | 96.44 | 87.62 | 45.29 | 59.77 | 29.13 | 87.61 |
| Nucleolar Equivalent Radius [μm] | 2.84 | 2.76 | 2.21 | 2.43 | 1.91 | 2.76 |
| Nucleolar 1^st^ Principal Axis [μm] | 5.24 | 5.39 | 4.57 | 4.96 | 3.74 | 5.71 |
| Nucleolar 2^nd^ Principal Axis [μm] | 5.09 | 4.76 | 4.04 | 4.18 | 3.52 | 4.82 |
| Nucleolar 3^rd^ Principal Axis [μm] | 4.96 | 4.69 | 3.38 | 3.96 | 3.09 | 4.39 |
| Nucleolar Average RI | 1.41 | 1.40 | 1.41 | 1.39 | 1.40 | 1.39 |
| Nucleolar Standard Deviation RI | 0.01 | 0.01 | 0.01 | 0.01 | 0.01 | 0.01 |
| Nucleolar Minimum RI | 1.35 | 1.38 | 1.38 | 1.38 | 1.36 | 1.36 |
| Nucleolar Maximum RI | 1.43 | 1.41 | 1.44 | 1.41 | 1.43 | 1.42 |
| Nucleolar Dry Mass [pg] | 35.28 | 27.04 | 18.16 | 17.33 | 9.37 | 25.65 |
| Nucleolar-Nuclear Volume Ratio [%] | 3.65 | 3.40 | 3.66 | 3.30 | 3.34 | 3.63 |

**Supplementary References**

S1. Glowinski, R. & Neittaanmaki, P. Partial differential equations: modelling and numerical simulation. *Computational methods in Applied Sciences*, Vol. 16 (Springer, Dordrecht, Netherlands, 2008).

**Supplementary Movies**

**Supplementary Movie 1. Time lapse holographic phase-contrast observations of onion epidermal cells. Dehydration allows imaging the cell with enhanced phase-contrast.**

Top Left: Global normalized contrast, with the logistic fitting overlapped in red. Top right: Differential global normalized contrast, whose peak marks the breaking plasmolysis event. The vertical lines correspond to the plasmolysis times, i.e., *t_P1_* = 12.9 h for the first cell, *t_P2_* = 12.2 h for the second cell, and *t_P3_* = 10.5 h for the third cell. For each cell, the temporal trend of the percentage ratio between the protoplast area, *A_P_*, and the cell area, *A_C_*, are shown with a linear fitting overlapped in red. After plasmolysis, detachment of the cell membrane from the cell wall is apparent and quantified here.

**Supplementary Movie 2: Finite element DNS of cell dehydration at increasing values of time before plasmolysis.**

The dehydration process generates curved liquid streamlines, displayed in red. The cell undergoing dehydration, before plasmolysis, reduces its height along the *z*-direction.

**Supplementary Movie 3: Finite element DNS of cell dehydration at increasing values of time after plasmolysis.**

The dehydration process generates curved liquid streamlines, displayed in red. The cell undergoing dehydration, after plasmolysis, reduces its height and its lateral sizes along the *x*- and *y*-directions.

**Supplementary Movie 4: Observing nucleus movements induced by dehydration.**

3D tracking of the nuclei for the three cells under test. Top right: temporal trend of the nuclear area in the *xy*-plane. Bottom right: temporal trend of the nuclear orientation with respect to the *x*-axis. Vertical dashed lines highlight the plasmolysis event for of the analyzed cells. Rotation of the three nuclei is observed much before plasmolysis.

**Supplementary Movie 5: Method to recover the rolling angles of the cells’ nuclei, to be exploited for 3D Phase Contrast Tomography (PCT).**

Top left: global normalized contrast. Vertical black lines denote the time interval in which the nucleus rotations are exploited for PCT, which is before the plasmolysis event (vertical violet line).

**Supplementary Movie 6: 3D tomographic reconstructions of two plant cells’ nuclei.**

First part: slice-by-slice visualization of the 3D RI tomograms. Second part: iso-levels representations with the nucleolus highlighted in red, along with the histograms of the RI distribution of the nucleus (yellow) and the nucleolus (red).

**Supplementary Movie 7: 3D tomographic reconstructions of four plant cells’ nuclei captured under red laser light.**

A QPM time lapse is shown, with colored boxes marking the reconstructed nuclei. For each nucleus, a slice-by-slice visualization of the 3D RI tomograms is shown. In the second part, iso-levels representations with the nucleolus highlighted in red are shown, along with the histograms of the RI distribution of the nucleus (yellow) and the nucleolus (red).

**Supplementary Movie 8: Time-lapse QPMs of onion epidermal cells captured under 20× and 10× magnification.**

Two QPMs time lapses are shown, which exhibit a large percentage of nuclei experiencing rotation with maximum rolling angle larger than 90 °, i.e. the percentage of nuclei that can be used for PCT.
